# Modeling human perception of orientation in altered gravity

**DOI:** 10.3389/fnsys.2015.00068

**Published:** 2015-05-05

**Authors:** Torin K. Clark, Michael C. Newman, Charles M. Oman, Daniel M. Merfeld, Laurence R. Young

**Affiliations:** ^1^Man Vehicle Laboratory, Department of Aeronautics and Astronautics, Massachusetts Institute of TechnologyCambridge, MA, USA; ^2^Jenks Vestibular Psychology Laboratory, Department of Otology and Laryngology, Massachusetts Eye and Ear Infirmary, Harvard Medical SchoolBoston, MA, USA; ^3^National Aerospace Training and Research CenterSouthampton, PA, USA

**Keywords:** orientation perception, hyper-gravity, vestibular, mathematical model, observer

## Abstract

Altered gravity environments, such as those experienced by astronauts, impact spatial orientation perception, and can lead to spatial disorientation and sensorimotor impairment. To more fully understand and quantify the impact of altered gravity on orientation perception, several mathematical models have been proposed. The utricular shear, tangent, and the idiotropic vector models aim to predict static perception of tilt in hyper-gravity. Predictions from these prior models are compared to the available data, but are found to systematically err from the perceptions experimentally observed. Alternatively, we propose a modified utricular shear model for static tilt perception in hyper-gravity. Previous dynamic models of vestibular function and orientation perception are limited to 1 G. Specifically, they fail to predict the characteristic overestimation of roll tilt observed in hyper-gravity environments. To address this, we have proposed a modification to a previous observer-type canal-otolith interaction model based upon the hypothesis that the central nervous system (CNS) treats otolith stimulation in the utricular plane differently than stimulation out of the utricular plane. Here we evaluate our modified utricular shear and modified observer models in four altered gravity motion paradigms: (a) static roll tilt in hyper-gravity, (b) static pitch tilt in hyper-gravity, (c) static roll tilt in hypo-gravity, and (d) static pitch tilt in hypo-gravity. The modified models match available data in each of the conditions considered. Our static modified utricular shear model and dynamic modified observer model may be used to help quantitatively predict astronaut perception of orientation in altered gravity environments.

## Introduction

Astronauts experience a series of altered gravity environments during space exploration missions: hyper-gravity during launch and re-entry, microgravity while on orbit or in transit, and hypo-gravity if landing on the moon or in the future on Mars. It is well-known that altered gravity affects sensorimotor function (Young et al., 1984; Parker et al., 1985). However, the effect of altered gravity on orientation perception remains poorly quantified. For example, astronauts often anecdotally report a sensation of tumbling upside down, or an “inversion illusion,” upon initial exposure to microgravity (Oman et al., 1986; Paloski et al., 2008), but to our knowledge these perceptions have not been quantified.

In addition, mathematical models of dynamic orientation perception are limited to normal Earth 1 G environments. To consider the effect of altered gravity in mathematical models of orientation perception, we first focus on hyper-gravity (i.e., >1 Earth G normally experienced) and then considering hypo-gravity (i.e., <1 Earth G). Since many hyper-gravity experiments use centrifugation, here and throughout we use “G” to refer to the net gravito-inertial force (GIF), or the combination of gravity and linear acceleration. Since by Einstein's equivalence principle forces of gravity and acceleration are ambiguous, we often refer to the net GIF level as the “gravity level.” One-G is equal to the 9.81 m/s^2^ of gravitational acceleration regularly experienced on Earth.

Previous experimental efforts have focused on perception of *static* tilts in hyper-gravity in the dark (Noble, 1949; Colenbrander, 1963; Schone, 1964; Miller and Graybiel, 1966; Schone and Parker, 1967; Schone et al., 1967; Correia et al., 1968; Cohen, 1973; Chelette et al., 1995; Jia et al., 2002). In most of these studies, a short-radius centrifuge was used to create a hyper-gravity environment and then subjects reported their static roll tilt perceptions using a subjective visual vertical (SVV) task (Aubert, 1861). Subjects typically overestimated their roll tilt angle in hyper-gravity.

For pitch tilt perception, other studies found that hyper-gravity caused a perception of being pitched nose up when the actual pitch angle was <30° forward (Schone, 1964; Correia et al., 1968; Cohen, 1973). When pitched nose down by roughly 30°, perception was unaffected by hyper-gravity. At this orientation, the approximate plane of the utricular component of the otolith organs is roughly perpendicular to the increased stimulation in hyper-gravity (Corvera et al., 1958; Curthoys et al., 1999).

To explain these results, there have been several models proposed for static orientation perception in hyper-gravity (Schone, 1964). First, Schöne hypothesized perceived tilt (e.g., pitch or roll) to be proportional to the shear force stimulation in the utricular plane. We note that the concept of a utricular “plane” is a simplification, since the utricular maculae are actually three-dimensional surfaces. For roll tilt, this relationship is given in Equation (1), where θ is roll tilt angle (either perceived or actual), *G* is the magnitude of the gravitational environment, or GIF, in Earth G's, and *K* is the proportionality constant.

(1) $$\theta_{per}=K*G*sin(\theta_{act})$$

The proportionality coefficient was initially estimated as 64°/G of shear force stimulation based upon pitch perception measures (Schone, 1964), however later data for roll tilt appear to support an estimate of 50–60°/G (Schone and Parker, 1967; Schone et al., 1967). Here we fix *K* = 60°/G. Correia et al. (1968) found the “utricular shear hypothesis” to be a poor fit; specifically, different combinations of angle and gravity level which yielded the same utricular shear force [G^*^sin(θ)] were perceived as different angles of tilt. A “tangent model” was found to be a better empirical fit (Equation 2) (Correia et al., 1968).

(2) $$\theta_{per}=\text{atan}\left(G*\tan\left(\theta_{act}\right)\right)$$

They hypothesized that the tangent model accounted for the “utricular compression component” influencing the otolith response. Alternatively Schöne et al. proposed the utricular shear hypothesis remains valid, but that it approaches a physiological limit at shear force magnitudes >1 G (Schone and Parker, 1967; Schone et al., 1967; Ormsby and Young, 1976).

Mittelstaedt proposed another model (Mittelstaedt, 1983a,b) for static tilt perception which postulated perception was driven by two distinct entities: graviceptor (e.g., otolith) cues and an “idiotropic vector” which draws perceptual reports toward the subject's body axis. The model was originally proposed to explain perceptual biases in 1 G (i.e., A- and E-effects) (Aubert, 1861; Muller, 1916), however by incorporating the complexities of non-linear transduction (Fernandez and Goldberg, 1976b) and the pitched up morphology of the utricle and saccule (Corvera et al., 1958; Curthoys et al., 1999), the model can produce overestimation in hyper-gravity. See the Appendix of Clark et al. (2015) for details on model implementation. Building upon the concepts of non-linear otolith function, Dai and colleagues proposed a model to predict tilt perception over a range of altered gravity levels and orientations (Dai et al., 1989). More recently, a model was developed to predict static orientation perception in altered gravity environments using otolith and tactile cues (Bortolami et al., 2006).

However, each of these models only considers *static* tilts in hyper-gravity. Several mathematical models have been proposed for *dynamic* orientation perception, as reviewed by Macneilage et al. (2008). Concepts from engineering estimation and control theory have been employed such as Kalman filters (Borah et al., 1988), extended and unscented Kalman filters (Selva, 2009), and particle filters (Laurens and Droulez, 2007; Karmali and Merfeld, 2012). In the integration of cues from the semicircular canals and otolith organs, it has been hypothesized that the central nervous system (CNS) employs internal models (Merfeld et al., 1999; Green and Angelaki, 2004) as well as an understanding of three-dimensional rotations (Glasauer, 1992; Holly and McCollum, 1996; Holly et al., 2011).

One of the better validated models is the “observer”-family of models (Merfeld et al., 1993; Merfeld and Zupan, 2002; Zupan et al., 2002; Vingerhoets et al., 2007, 2009; Newman, 2009; Rader et al., 2009), which have been used to predict a wide range of illusory perceptions. The model is based upon the “observer” concept from estimation theory (Kalman, 1960; Kalman and Bucy, 1961; Luenburger, 1971) which uses an internal model to predict and evaluate feedback measurements (Oman, 1982, 1990). While effective for a wide range of motion stimuli in 1 G, the observer models do not predict overestimation in hyper-gravity, even for static tilts. Instead, for any altered gravity environment the observer model predicts near veridical perceptions of tilt, limiting their application to a 1 G environment. However, we recently proposed a modification to the observer model, which allows for it to predict the static and dynamic overestimation of roll tilt experimentally observed across a range of conditions (Clark et al., 2015). The modification is based upon the hypothesis that the CNS treats otolith stimulation in the utricular plane different than stimulation out of the utricular plane.

In the remainder of this paper, first in the methods we detail our two modified models: the modified utricular shear model for *static* orientation perception and the modified observer model that can make predictions for *static and dynamic* orientation perception. In the results, data for static roll tilt perception in hyper-gravity are compared to previous mathematical models. Finding systematic errors between the previous models and roll tilt perception in hyper-gravity, we next compare to our modified utricular shear model and modified observer model.

We then transition to pitch tilt perception in hyper-gravity, comparing the modified observer and modified utricular shear models to previous available data. As a novel contribution of this paper, we show the modified observer model predictions for pitch tilt in hyper-gravity which emphasizes the criticality of the hypothesized differential weighting in the pitched-up utricular plane.

Finally, the modified models are simulated in hypo-gravity environments, including lunar and Martian gravity levels. Novel model predictions are first made for roll tilt and then for pitch tilt, across a range of tilt angles and hypo-gravity levels.

To summarize the various models considered and their performance in each different condition (static vs. dynamic tilts, 1 G vs. hyper-G vs. hypo-G, and roll vs. pitch) we provide Table 1 as reference. We note that none of the models considered make meaningful predictions in microgravity (i.e., 0 G), where “orientation” is no longer relative to the direction of gravity, and thus this altered gravity environment is omitted from Table 1. We also do not consider yaw tilts (e.g., supine subject in a bbq-style rotation) or combinations of different axes. The shaded boxes denote specific conditions considered in the current paper.

**Table 1:**
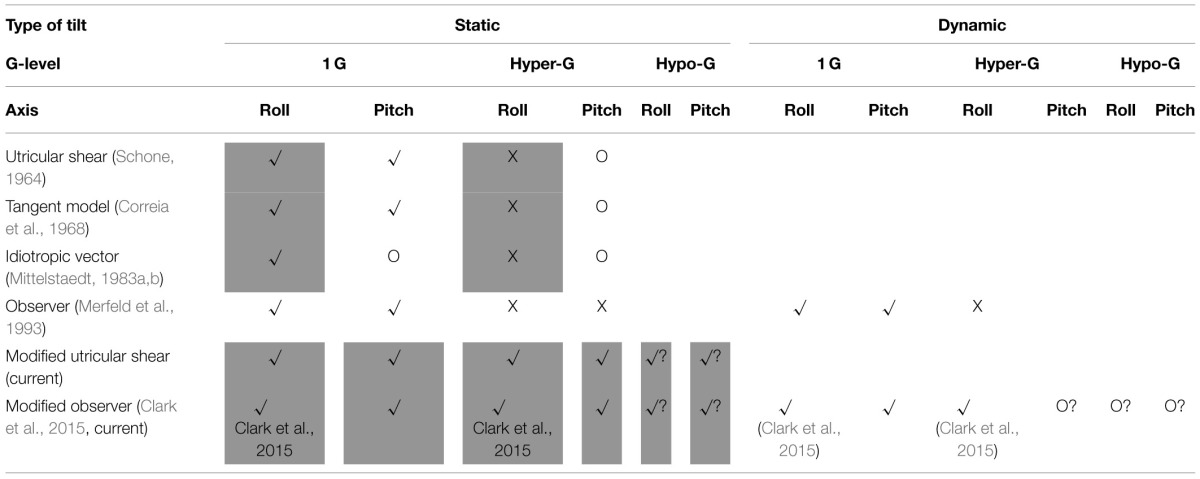
**Summary of previous and current models for orientation perception in altered gravity**.

*√, Quality fit of the data; X, systematic errors in fitting the available data; O, model can make predictions but either the original authors do not present them or we do not consider them here; O?, model can make quantitative predictions but they are not presented here and have not yet been experimentally validated; √surd?, model makes predictions which we present here but have not yet been experimentally validated; empty, model cannot make predictions or we would not expect the predictions to be valid; gray shaded, presented in the current paper*.

It is well-known that exposure to altered gravity drives sensorimotor adaptation and a reinterpretation of sensory orientation cues (Young et al., 1984; Parker et al., 1985). In fact, we recently observed less dynamic overestimation of roll tilt in hyper-gravity on a second presentation (Clark et al., 2015). However, like almost all earlier sensory integration models of spatial orientation, the models considered here do not have adaptive mechanisms to reproduce this effect, so we will only aim to model perception on initial exposure to an altered gravity environment.

## Materials and methods

We recently completed an experiment studying roll tilt perception in hyper-gravity (Clark et al., 2015). In this experiment, subjects reported roll tilt perception using a haptic task, in which they aligned a hand-held bar with their perceived horizontal (Wade and Curthoys, 1997; Bortolami et al., 2006; Park et al., 2006). We measured at roll tilts of −20, 10, 20, and 40° (by our convention positive angles corresponded to tilts to the left; however we found no evidence of left/right asymmetries) and net gravito-inertial levels (G-levels) of 1, 1.5, and 2 G's. This previous experiment was approved by the Environmental Tectonics Corporation/NASTAR Center's Internal Review Board and MIT's Committee on the Use of Humans as Experimental Subjects. Using this dataset, here we evaluate several previously proposed models for static orientation perception in hyper-gravity. Specifically, we consider the utricular shear model (Schone, 1964; Schone and Parker, 1967; Schone et al., 1967), tangent model (Correia et al., 1965, 1968), and Mittelstaedt's idiotropic vector model (Mittelstaedt, 1983a,b). To differentiate our recent dataset from other experiments studying static tilt perception in hyper-gravity, we refer to this study as our “current study.”

### Modified utricular shear model

**Figure 1:**
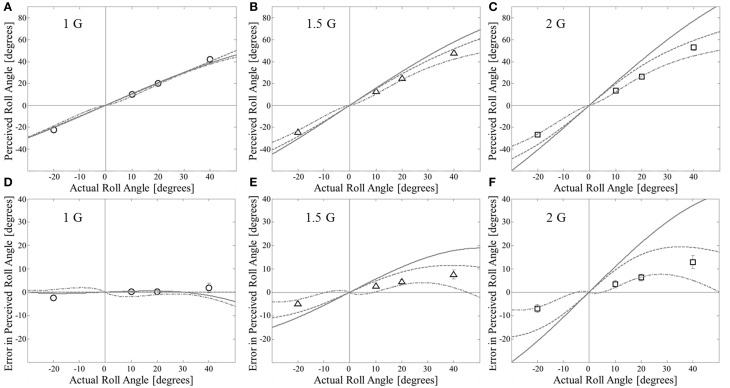
**Comparison of previous static models for hyper-gravity roll tilt perception to experimental data**. Data are means ± 1 SE (*N* = 48 per point). Utricular shear model (solid line) uses *K* = 60°/G. The tangent model is shown as the dotted line. The Mittelstaedt model (dash-dot line) uses all parameters as defined in Mittelstaedt (1983a). **(A**–**C)** Show perceived roll angle; **(D**–**F)** Show the same information but as error in perceived roll angle (perceived–actual). By our convention positive angles are tilts to the left.

As will be seen (Figure 1) prior models do not fit our current study data well (static roll tilt in hyper-gravity). Alternatively we propose a “modified utricular shear” model. The model is empirical and *ad hoc*, but we provide some justification here. There is evidence showing the change in the otolith afferent firing rates are approximately proportional to the force acting along the neuron's polarization direction in monkeys (Fernandez and Goldberg, 1976a,b,c). Hence it was logical for the proposed model to be of the form G^*^sin(θ), since that is the physical quantity causing changes in firing rates. On a micro-level, θ may refer to the angle between the gravity force and an individual neuron's polarization direction. However, at a population level, θ may refer to the roll angle for example, where each neuron's gain is proportional to how closely its polarization direction is aligned with stimulation from roll tilt. Thus, we began with the traditional utricular shear model (Equation 1), but rearranged it into 1 G and hyper-G terms and then added an additional free parameter (*M*) to the hyper-gravity term. This model allows for the 1 G and hyper-gravity perceptions to be fit separately, unlike the traditional utricular shear model. However, both hyper gravity levels across all angles still must be fit with a single free parameter. We fit the model to our current dataset (Clark et al., 2015), using a hierarchical regression with subject as the identifier. Model fit parameters are provided in Table 2.

(3) $$\theta_{per}=\rho_{i}+K*\sin\left(\theta_{act}\right)*\left[1+M*(G-1)\right]$$

**Table 2:** **Modified utricular shear model for static roll tilt**.

**Coefficient**	**Units**	**Estimate**	**Standard error**	**Z-values**	***p*-Values**
ρ_*i*_	Degrees (°)	−0.29	0.83	−0.34	0.73
*K*	Degress/G (°/G)	64.6	1.53	42.1	<0.0005
*M*	Unitless	0.26	0.035	7.48	<0.0005

In Section Comparison of Static Pitch Tilt in Hyper-Gravity to Modified Utricular Shear and Modified Observer Model, the modified utricular shear model is compared to previous pitch tilt perception data in hyper-gravity. To make this comparison, we must account for the pitched up orientation of the utricular plane (θ_utricule_). This is done as in the traditional utricular shear model and the resulting formulation for pitch (δ) is provided in Equation (4).

(4) $$\delta_{per}=K*\sin\left(\delta_{act}+\theta_{utricule}\right)*\left[1+M*\left(G-1\right)\right]-\theta_{utricule}$$

The pitched up angle of the utricular plane (θ_utricule_) is defined in Table A2 in Supplementary Material. In the application of the modified utricular shear model to pitch tilt perception (**Figures 3, 6, 7**), the fitted parameters (*K*, *M*) are taken from the roll tilt fits and applied directly.

### Modified observer model summary

We recently proposed a modification (Clark et al., 2015) to a previously proposed model for dynamic orientation perception (Merfeld et al., 1993; Merfeld and Zupan, 2002). Details of the model and the modification are provided in the Supplementary Appendix. These details are particularly critical for the complexities of the pitch tilt simulations included herein. In brief, we build upon the hypothesis from Clark et al. (2015) that linear acceleration feedback errors are differentially weighted whether they are in the utricular plane or perpendicular to it. Here we consider the implications of the utricular plane being pitched up relative to the head level orientation. The utricular orientation becomes relevant for pitch tilt perceptions in altered gravity. The modified model was evaluated in a series of altered gravity environments and the model predictions were compared to experimental data when available. We emphasize that the observer model can predict orientation perception during *dynamic* motions and in fact matches experimental perceptions of dynamic roll tilt in hyper-gravity (Clark et al., 2015). However, to our knowledge there is not quantitative data for *dynamic* perception of orientation in other altered gravity paradigms (e.g., pitch tilt, hypo-gravity, etc.). Thus, here we simulate the modified observer model and calculate *static* perceptions (details below) in two novel paradigms. The model was simulated with static pitch tilt in hyper-gravity and compared to previous studies (Correia et al., 1968; Cohen, 1973). Finally, the model was simulated with static roll tilt and static pitch tilt in various hypo-gravity environments to make quantitative hypotheses for future experimentation.

## Results

### Comparison of static roll tilt in hyper-gravity to previous models

Previous models for static roll tilt perception are often compared to data by plotting perceived angle vs. actual angle. We use this approach to compare our experimental data (Clark et al., 2015) to model predictions for the utricular shear model (Schone, 1964), tangent model (Correia et al., 1968), and Mittelstaedt's idiotropic vector model (Mittelstaedt, 1983a) in Figures 1A–C (Figure 1A = 1 G, Figure 1B = 1.5 G, and Figure 1C = 2 G). However, the perceived angle in any condition is primarily determined by the actual angle, making the additional effects of hyper-gravity and the specific model difficult to observe when plotted in this format. Thus, we also plot the *error* in the perceived angle (perceived–actual angle) as a function of the actual angle. The comparisons between the three previous static models and our experimental data are also provided in the error format in Figure 1 (Figure 1D = 1 G, Figure 1E = 1.5 G, and Figure 1F = 2 G).

All three models approximately fit the dataset in 1 G across the angles tested; however none of the models appropriately explains the perceptions observed in hyper-gravity. This is accentuated when viewing the perceptual errors (Figures 1D–F). In particular, both the utricular shear and tangent model predict much greater overestimation in hyper-gravity than was measured. In the utricular shear model, the free “*K*” parameter (Equation 1) can be reduced to better fit the hyper-gravity static perceptions. However, this can only be done at the expense of incorrectly predicting the 1 G responses. Specifically, a smaller *K* parameter (Equation 1) leads to the utricular shear model predicting substantial underestimation of roll tilt in 1 G that is inconsistent with the near veridical perceptions observed. To quantify the quality of the fits between each of the models and the current data for roll tilt in 1, 1.5, and 2 G's the coefficient of determination (*R*^2^) was calculated between the model predictions and the mean responses, in terms of perceptual errors, across subjects for each angle and gravity level combination. For the tangent model *R*^2^ = 0.06 and for the utricular shear model *R*^2^ = −2.8 (negative values correspond to the model fitting the data worse than the global mean), further confirming the poor fits.

The Mittelstaedt model does better, approximately fitting the current dataset in hyper-gravity for small tilt angles (10 and 20°). However, the model predicts a decreased amount of overestimation for larger angles (e.g., 40°). Yet the overestimation in hyper-gravity that we previously observed at 40° tilt is significantly larger than at 10 or 20°. Thus, the “shape” of the Mittelstaedt model, particularly when viewing the perceptual errors, does not match the experimental data well. The coefficient of determination for the Mittelsatedt model was *R*^2^ = 0.57. It should be mentioned, and will be shown later, that the lack of fit is not an issue with the current dataset (Clark et al., 2015) being in disagreement with previous datasets (Colenbrander, 1963; Schone, 1964; Miller and Graybiel, 1966; Correia et al., 1968) upon which these models were developed. In fact, this dataset matches previous datasets quite well considering the differing methodologies (SVV vs. haptic task). Instead the previous data only appears to fit the previous models relatively well when viewing perceived angle, which is dominated by the change in actual angle, as opposed to error in perceived angle.

### Comparison of static roll tilt in hyper-gravity to modified utricular shear and modified observer model

Since the previously proposed models fail to sufficiently explain the overestimation measured in hyper-gravity, we propose an alternative model, the modified utricular shear model (Equation 3). The model is fit to our current dataset (Clark et al., 2015), using a hierarchical regression with subject as the identifier and the results are provided in Table 2.

For small angles, to achieve an accurate perception in 1 G, the *K* coefficient should be 57.3°/G (180/π). Our fit has a slightly larger estimate (64.6°/G) which yields slight overestimation at small angles, but less underestimation at larger angles in 1 G. The *K* coefficient estimate is very similar to a previous traditional utricular shear fit of 64°/G (Schone, 1964).

**Figure 2:**
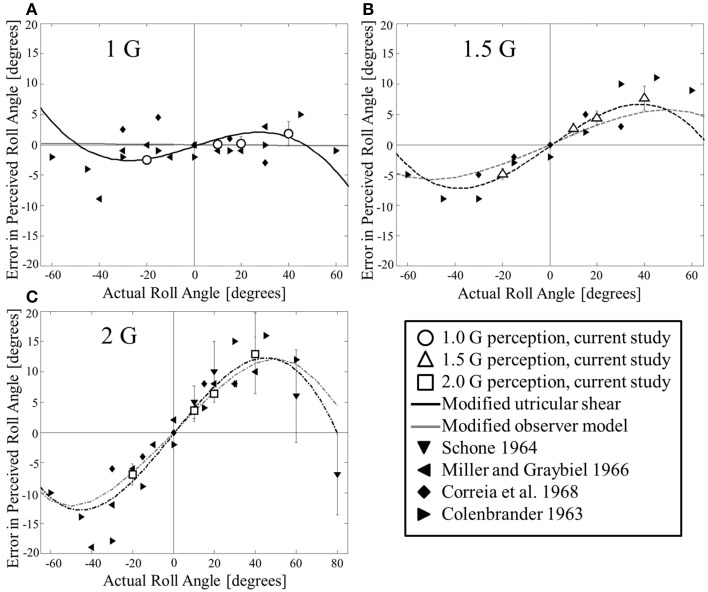
**Comparison of current and previous static roll tilt perception to modified utricular shear model and modified observer model predictions**. Comparisons are made at **(A)** 1 G, **(B)** 1.5 G, and **(C)** 2 G. Many of the previous studies only provide means, in which case no error bars are included here.

The estimated value of *M* = 0.26 implies that the overestimation seen in hyper-gravity is only about 26% of that which would be expected from the traditional utricular shear model. The model fits the current data quite well-across all of the gravity-levels and angles tested. It also, at least qualitatively, fits data from many of the previous SVV experiments well, as seen in Figure 2 (black lines). Here we focus exclusively on errors in perceived roll tilt to accentuate any differences between the model fit and the experimental data. The coefficient of determination for the modified utricular shear model was *R*^2^ = 0.97, a dramatic improvement upon previous models (only data from the current dataset were included in the *R*^2^ calculation to allow for direct comparison to the *R*^2^-values for previous models).

Prior experiments used a different psychophysical task for measuring perceived roll (i.e., SVV), different motion devices, and tested at larger angles than the current dataset to which the proposed modified utricular shear model was fit. The match between the model predictions and available data provides support that the model empirically predicts static roll perceptions over a large range of angles and hyper-gravity levels.

The modified observer model was previously fit to the current data for roll tilt in hyper-gravity (Clark et al., 2015). However, for comparison, Figure 2 overlays the modified observer model predictions (gray lines) with the modified utricular shear model and other datasets. Across the angles and hyper-gravity levels considered, the two models mimic each other substantially and therefore both match the available data quite well. For the modified observer model *R*^2^ = 0.93, indicating an excellent fit. While the coefficient of determination is slightly better for the modified utricular shear model than the modified observer model, we emphasize that the modified utricular shear model was directly fit to all of the current data while the added parameter from the modified observer model was fit to just one particular case (20° tilt in 2 G's, see Supplementary Appendix for details).

### Comparison of static pitch tilt in hyper-gravity to modified utricular shear and modified observer model

**Figure 3:**
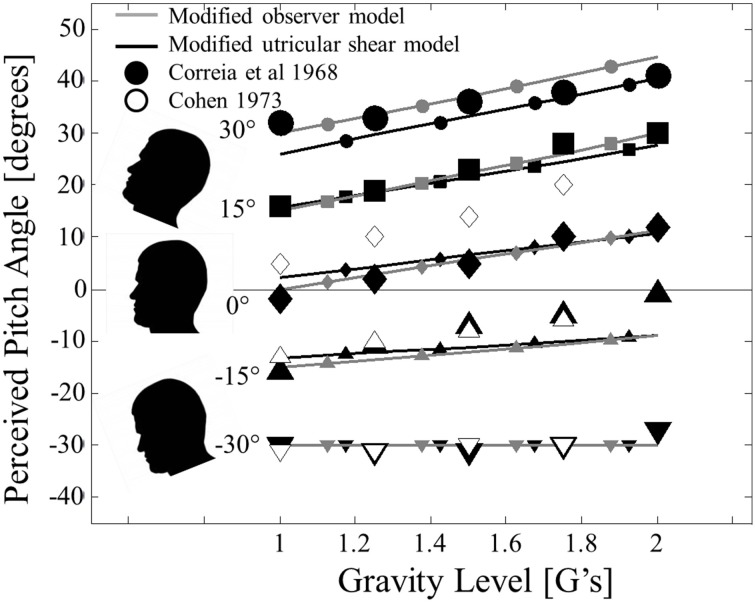
**Modified model predictions for static pitch tilt perception in hyper-gravity**. Modified observer model predictions (solid gray lines with small gray symbols) and modified utricular shear model (solid black lines with small black shapes) are compared to previous experimental reports from Correia et al. (1968) (filled black symbols) and Cohen (1973) (unfilled black symbols). Head pitch angle is signified by symbol shape: 30° (•), 15° (■), 0° (◊), −15° (▲), and −30° (▼). The plot is formatted to mimic Figure 1 of Correia et al. (1968). Error bars were not originally provided.

We now transition to static *pitch* tilt perception in hyper-gravity. Pitch perception errors in hyper-gravity are not symmetric about upright like roll errors. Specifically, pitch in hyper-gravity is characterized by perceiving oneself as being pitched nose up relative to actual orientation when upright, pitched up, or when pitched nose down by <30° (Correia et al., 1968; Cohen, 1973). To directly compare to the most complete hyper-gravic static pitch perception dataset (Correia et al., 1968), the modified observer and modified utricular shear models were simulated for pitch angles of −30, −15, 0, 15, and 30° (negative pitch angles correspond to nose down) and gravity levels of 1, 1.25, 1.5, 1.75, and 2 G's (Figure 3).

As desired, the modified observer and modified utricular shear models predict qualitatively different static perceptions for pitch than for roll. Whereas roll tilt perception is symmetric about upright (0° roll tilt), pitch perception is asymmetric. In particular, at upright (0° of pitch tilt) there is a noticeable effect of gravity; hyper-gravity produces a perception of being pitched nose up. Increasing hyper-gravity levels causing a sensation of nose-up pitch relative to the 1 G level is a trend that exists for all of the angles simulated except for −30° (pitched nose down). At this orientation, increasing gravity level has a negligible effect on the veridical pitch perception. Each of these characteristics is observed in the two previous experimental datasets (Correia et al., 1968; Cohen, 1973).

To quantify the quality of the fit between the models' predictions and the perceptions, we again calculate the coefficients of determination (*R*^2^). To match the analysis for roll tilt, these are calculated using the perceptual *errors* (Note that Figure 3 shows the perceived *angles* and predicted perceived *angles* and not the perceived *errors* to mimic the format of Figure 1 from Correia et al., 1968). Also note that the perceived angles in each previous dataset (Correia et al., 1968; Cohen, 1973) are estimated from the published figures, so these coefficients of determination are approximate. Both models fit the Correia et al., 1968 dataset quite well (*R*^2^ = 0.72 for the modified observer model and *R*^2^ = 0.65 for the modified utricular shear model). Remember that neither model is explicitly “fit” to these data; instead the models are fit to roll tilt in hyper-gravity and are now simply applied to pitch tilt in hyper-gravity. The model fits to the Cohen (1973) dataset are not quite as clean (*R*^2^ = 0.29 for the modified observer model and *R*^2^ = 0.45 for the modified utricular shear model). However, most of the lack of fit is due to an offset for upright perception (0° pitch) across each gravity level (unfilled diamonds in Figure 3). In fact, it would be impossible for any model to fit both the Correia et al. (1968) and the Cohen (1973) data well, since the two datasets diverge in this condition. The major effect of increasing levels of hyper-gravity causing an increasingly pitched nose up perception is observed in both models' predictions.

The asymmetry in the observer model's static pitch predictions, as well as those for the modified utricular shear model, can be attributed to the assumed utricular plane orientation. Only in orientations where increasing the gravity level modifies the stimulation of the otoliths in the utricular plane, will the perceptual response change with gravity level. For roll tilt, the null orientation where changes in gravity magnitude do not effect perception is upright. For pitch, nose down pitch equal to θ_utricle_ = 30° will yield accurate pitch perceptions even in hyper-gravity. Hence, the assumed orientation of the utricular plane is essential to the model's performance, including its asymmetry. It was assumed the plane was level in roll and pitched up 30° relative to the head fixed coordinate frame based upon morphological studies. The Correia et al. (1968) and Cohen (1973) data in pitch support the view that the perceptual asymmetry is tied to the utricular plane and thus also supports our assumption that the modified observer processing asymmetry originates in differential weighting of head fixed utricular vs. saccular information.

### Model predictions of static roll tilt in hypo-gravity

We now transition to considering the model predictions of orientation perception in hypo-gravity (i.e., gravity environments <1 Earth G). Since the previous models (utricular shear, tangent, and idiotropic vector models) have systematic errors in *hyper-gravity* roll tilt perception, we do not further consider them for *hypo-gravity*, where presumably they would also have systematic errors.

**Figure 4:**
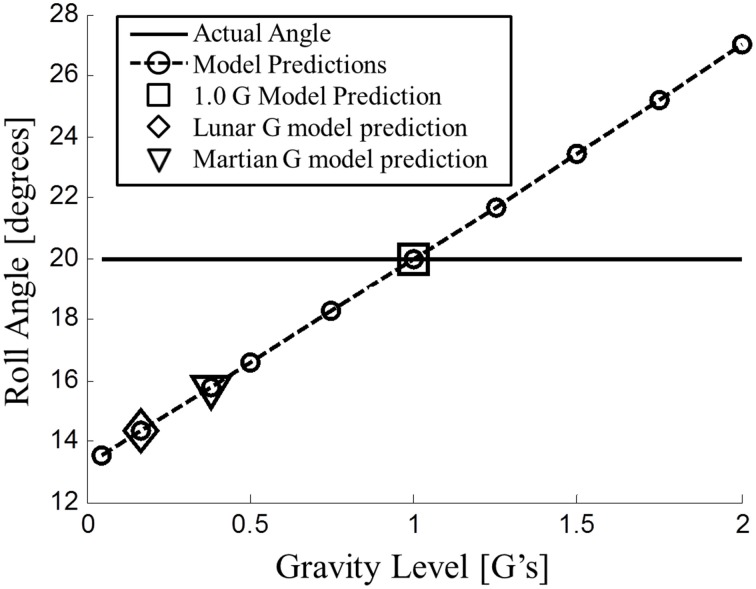
**Modified observer model predictions for static roll tilt perception across gravity levels**. At <1 G (hypo-gravity), the model predicts underestimation of roll tilt angle. Lunar (~1/6 G) and Martian (~3/8 G) are highlighted (diamond and triangle, respectively).

First, we focus on the modified observer model predictions in hypo-gravity for roll tilt perception. Without the modification detailed above, previous versions of the observer model predicted veridical static roll tilt perceptions in hypo-gravity. To test the modified observer model's predictions it was simulated with the example 20° static roll tilt at various gravity levels (Figure 4).

As intended, the modified observer model simulated the static overestimation in hyper-gravity and the near accurate static perception in 1 G (marked with a square in Figure 4). However, the modified model now makes a novel prediction: *underestimation* of static roll tilt in hypo-gravity (0 < $\left|\vec{g}\right|$ < 1). The amount of predicted underestimation was more extreme for lower gravity levels. Of particular interest are the lunar (~1/6 G) and Martian (~3/8 G) hypo-gravity levels, which are specially marked in Figure 4. At very low gravity levels (e.g., 0.05 G), the perception of the 20° roll tilt approaches ~13.2° or underestimation of ~34% of the actual angle. This amount of underestimation is similar to the amount of overestimation observed in 2 G. Note that simulating the model at exactly 0 G results in a singularity when the gravity vector is normalized by its magnitude, and was not simulated.

**Figure 5:**
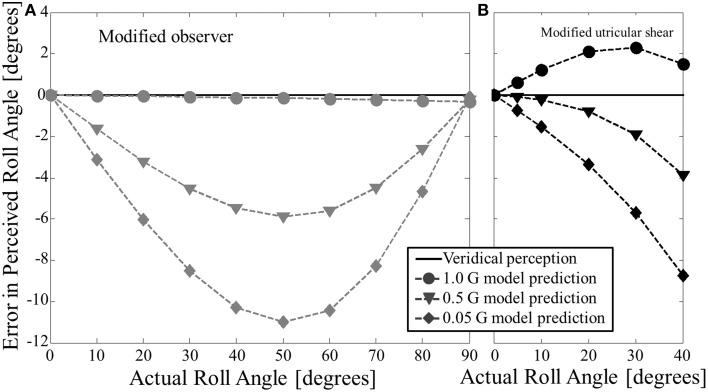
**Modified model predictions for static roll tilt perception in hypo-gravity. (A)** Shows the modified observer model predictions. **(B)** Shows the modified utricular shear model predictions. Both models predict underestimation of roll tilt (negative errors for positive tilt angles) for acute angles in hypo-gravity.

To provide quantitative hypotheses to allow for direct comparison with future experiments, we simulated the modified observer model for static roll tilt in hypo-gravity across a range of conditions. The modified observer model's predicted error in roll tilt (perceived–actual, as above) at 0.05, 0.5, and 1 G's across a range of angles is shown in Figure 5A.

The amount of underestimation predicted by the modified observer model depends upon both roll tilt angle and hypo-gravity level. The magnitude of underestimation peaks at approximately 50° of roll tilt for each case of hypo-gravity simulated. For a particular angle, the underestimation is roughly proportional to the difference in G-level between the hypo-gravity level and 1 G. Thus, 0.05 G yields roughly twice as much predicted underestimation as 0.5 G.

For comparison, Figure 5B shows the modified utricular shear model's predictions for static roll tilt in hypo-gravity. First, note that in 1 G (circles) the model predicts slight overestimation (same prediction shown in Figure 2A). However, in hypo-gravity (e.g., 0.5 G (triangles) and 0.05 G (diamonds)) the modified utricular shear model also predicts underestimation of roll tilt. The amount of underestimation is similar, though general less, for the modified utricular shear model. Since the modified utricular shear model was explicitly fit to hyper-gravity perception for angles no >40°, this model's predictions in hypo-gravity are only shown up to 40°. Unlike the modified observer model, the modified utricular shear model, if simulated at larger roll tilt angles (50–90°), predicts increasing underestimation (not shown).

### Model predictions of static pitch tilt in hypo-gravity

**Figure 6:**
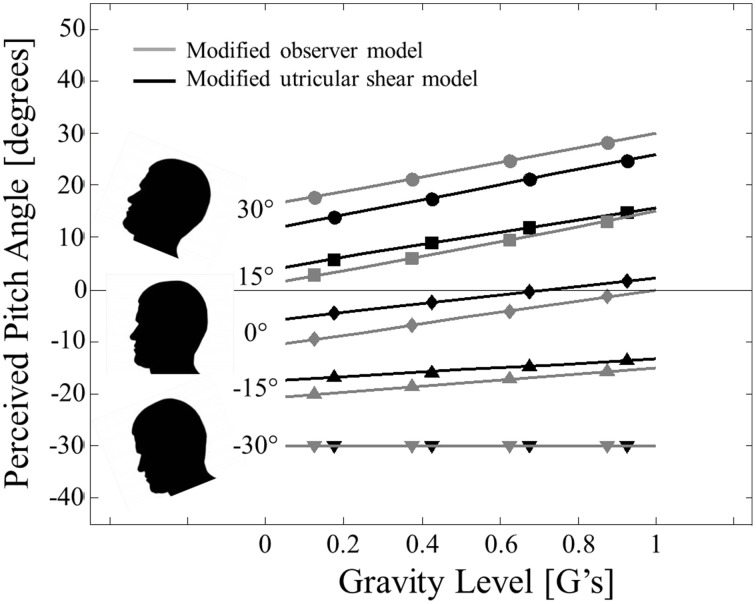
**Model predictions for static pitch tilt perception in hypo-gravity**. Modified observer model predictions (solid gray lines with gray symbols) and modified utricular shear model (solid black lines with black shapes) are presented. Head pitch angle is signified by symbol shape: 30° (•), 15° (■), 0° (◊), −15° (▲), and −30° (▼).

As a final novel prediction, the modified models are simulated for static pitch tilt in hypo-gravity. Specifically, we consider the same pitch tilt angles for hyper-gravity (−30, −15, 0, 15, and 30° of head tilt, where again negative pitch angles correspond to nose down), but now simulate at several hypo-gravity levels (0.05, 0.25, 0.5, 0.75 G) as well as at 1 G. The modified utricular shear and modified observer model predictions for static pitch tilt perception in hypo-gravity are presented in Figure 6 with gravity level on the ordinate (mimicking Figures 1, 3 from Correia et al., 1968).

First, the modified models predict nearly accurate perceptions in 1 G (far right of Figure 6, also shown in far left of Figure 3). However, going from right to left across Figure 6 shows that hypo-gravity causes a predicted perception of feeling pitched nose down relative to the actual angle. For example, the modified observer model simulated at 30° of nose up pitch (top gray line in Figure 6) in 1 G (far right end of line) shows an accurate pitch perception of 30°. However, at 0.05 G (far left end of top gray line) the modified observer model predicts a pitch perception of only 16.75° pitched nose up, or an error of −13.25° in which the simulated subject feels pitched nose down relative to their actual pitch angle. Note that in this example the simulated subject still feels pitched nose up (by 16.75°), just not as much as he/she actually is (30°).

In hypo-gravity these predicted perceptual errors persist until pitched nose down at −30°. At this orientation, as detailed previously for hyper-gravity, the utricular plane is perpendicular to the direction of gravity and the predicted perception in independent of the magnitude of gravity. The exact predictions for pitch perception in hypo-gravity vary slightly between the modified utricular shear and modified observer models. However, both modified models predict the major effect of perceptual errors of feeling pitched nose down relative to actual pitch angle in hypo-gravity.

**Figure 7:**
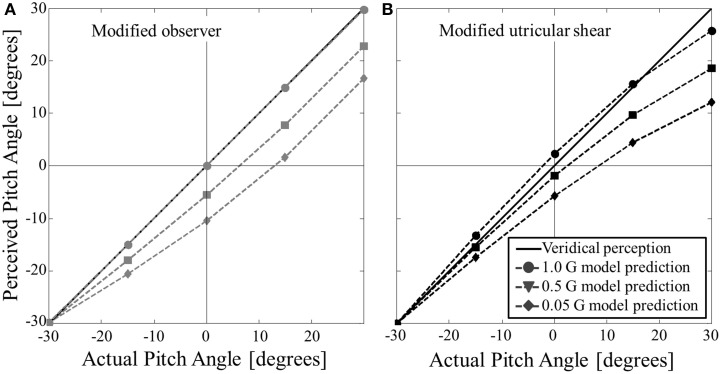
**Modified model predictions for static pitch tilt perception in hypo-gravity. (A)** Shows the modified observer model predictions. **(B)** Shows the modified utricular shear model predictions. The unity line (y = x) corresponds to accurate perception. Both models predict misperceptions of feeling pitched nose down relative to actual angle in hypo-gravity (predicted pitch perception is more negative than unity line). The exception is at an actual pitch angle of −30° (nose down), where perceptions are accurate at all hypo-gravity levels.

To further clarify the effect of pitch tilt angle in hypo-gravity the same simulation predictions from Figure 6 are plotted in Figure 7, now with angle of the actual pitch tilt on the abscissa. To mimic Figure 5 (roll tilt in hypo-gravity), here we only consider hypo-gravity levels of 0.05, 0.5, and 1 G. Note that the ordinate shows the perceived pitch angle, and not the error in perceived pitch angle, to more clearly show the direction of the predicted perceptual errors.

## Discussion

We considered several models for tilt perception in altered gravity. First, the previously proposed utricular shear, tangent, and idiotropic vector models were unable to fit measured hyper-gravity static roll tilt perceptions. We proposed a modified version of the utricular shear model for static roll tilt perception that not only matched our recent dataset to which it was fit (Clark et al., 2015), but qualitatively fit previous results across a wide range of conditions. To address dynamic perception in altered gravity we recently proposed a modification to the observer model, detailed herein. The modification was based upon the hypothesis that the CNS weights errors in expected otolith sensory signals differentially whether they are in or perpendicular to the utricular plane. We further demonstrate that the modified observer model is able to predict roll tilt perceptions in hyper-gravity across the range of conditions considered. By assuming the utricular plane is pitched up by approximately 30° relative to the head horizontal plane, the modified observer model was able to match the available experimental perception data for static pitch tilts in hyper-gravity. Making a similar assumption about utricular plane orientation allowed for the modified utricular shear model to match data for static pitch tilt in hyper-gravity. Finally, we simulated the modified utricular shear and modified observer models for static roll tilt and static pitch tilt in hypo-gravity, making quantitative predictions across a range of conditions.

### Previous models, modified utricular shear model, and modified observer model for static roll tilt in hyper-gravity

The current data could not be fit well-with any of the previously proposed models we considered (utricular shear, tangent, and Mittelstaedt's “idiotropic vector” model). The failures of these models to quantitatively fit the current data were primarily due to incongruences between the models and the data as opposed to the current data and previous SVV hyper-gravity roll tilt perception data (Colenbrander, 1963; Schone, 1964; Miller and Graybiel, 1966; Correia et al., 1968), which generally match quite well (a quantitative comparison is provided in Figure 2). The utricular shear and tangent models were previously only compared to data in terms of perceived angle vs. actual angle, which masks the effect of hyper-gravity with the variation in angle. When we compared to data in terms of perceptual *errors* (perceived–actual angle), extenuating the effect of hyper-gravity, quality of the fit becomes more evident (Figures 1D–F). Mittelstaedt's model was previously only qualitatively compared to the observed effect of hyper-gravity on roll tilt perception (Mittelstaedt, 1983a).

We proposed a modified version of the utricular shear model that, with two free parameters, not only fit the current data across three gravity levels and four angles we tested (Clark et al., 2015), but also qualitatively fit previous data even at gravity and angle combinations which the model was not specifically trained upon. The model is a simple empirical fit, but does indicate that the amount of overestimation in hyper-gravity is only about 26% of that expected from the traditional utricular shear model. As to the underlying physiological explanation for this reduction in overestimation of roll tilt in hyper-gravity, we can only speculate. We hypothesize it may be due to the CNS utilizing information from other static graviceptors (e.g., otolith cues out of the utricular plane, proprioceptive, tactile, somatosensory, or potentially trunk graviceptors).

We recently modified an existing, dynamic, canal–otolith interaction model with the hypothesis that the CNS treats otolith stimulation in the utricular plane differently than that out of plane. The modified observer model was previously considered for static roll tilt in hyper-gravity (Clark et al., 2015). Here we extend the comparison to a wider range of roll tilt angles and find the modified observer model matches the available data quite well (Figure 2).

### Modified utricular shear and modified observer models for static pitch tilt in hyper-gravity

For *roll* tilt perception in hyper-gravity, as previously considered (Clark et al., 2015), the importance of pitched-up orientation of the utricular plane is not explicitly apparent. Specifically, the differential weighting could occur between the head horizontal plane (x–y) and vertical direction (z) and the model predictions for roll tilt would be unaffected. This is because in roll tilt the otolith shear stimulus is in the direction of both the y and y' axes, which are aligned.

The criticality of the differential weighting being in the utricular plane becomes apparent when considering *pitch* tilt perception in hyper-gravity. Here the shear stimulus is in the direction of the x' axis and the x' and x axes are misaligned by 30°. Matching the available experimental data (Correia et al., 1968; Cohen, 1973), the model predicts a perception of being pitched nose up relative to the actual pitch angle in hyper-gravity (Figure 3). The exception to this is for pitched nose down orientations of at least 30°. At this orientation, the utricular plane (pitched up relative to head-level by approximately 30°) is aligned perpendicularly with the increasing GIF; hyper-gravity causes compressive forces to the utricular membrane as opposed to additional utricular shear.

Data from Correia et al. (1968) and Cohen (1973) do not provide standard errors to their measures. However, the two independent data sets are in close agreement (Figure 3) and Schone (1964) shows a similar effect of hyper-gravity on static pitch perception. In Correia et al. (1968) and Schone (1964) whole-body tilts were performed, while in Cohen (1973) the tilts were head-on-body suggesting that proprioception in the neck is not the primary cause of the pitch perception asymmetry in hyper-gravity. The Correia et al. (1968) and Cohen (1973) data sets do differ when the subject is upright (Figure 3), but only by an offset that is independent of gravity level; the effect of hyper-gravity causing a pitch nose up perception is similar between the studies. Together these datasets are consistent with the hyper-gravity pitch predictions from the observer model with the hypothesis that the CNS treats otolith stimulation in the utricular plane (pitched up by 30°) differently than out of plane stimulation. By making a similar assumption about the pitched up orientation of the utricular plane, the modified utricular shear model was able to predict the available data for static pitch tilt in hyper-gravity.

### Modified utricular shear and modified observer models for static roll and pitch tilt in hypo-gravity

Finally, the modified observer model and modified utricular shear model were simulated with static roll tilt in hypo-gravity leading to a novel prediction: underestimation of roll tilt in hypo-gravity. For the modified observer model, the amount of underestimation was greater for more extreme (smaller) hypo-gravity levels, and peaked at approximately 45–50° of roll tilt. The modified utricular shear model also predicted underestimation in hypo-gravity with more underestimation at more extreme hypo-gravity levels. We only present the modified utricular shear model predictions up to 40° to stay within the angle limits to which the model was fit in hyper-gravity (Figure 5B). Predictions for roll tilt angles >40° may be considered outside of the scope of the modified utricular shear model.

To our knowledge there have been two attempts at quantifying static roll perception in hypo-gravity (Dyde et al., 2009; De Winkel et al., 2012), but neither directly address the predictions in Figures 4, 5. In the experiments, subjects only reported perceptions when upright (roll = 0°) or on their side (roll = +90 or −90°). At upright, the model predicts accurate upright static perception independent of gravity level, in agreement with the hypo-gravity experiments. Similarly, at 90° of roll tilt, the model prediction of static perception is accurate across the range of hypo-gravity levels. Only at acute angles of roll tilt do the modified models predict underestimation of static roll tilt in hypo-gravity. Future experiments should test a wide range of hypo-gravity levels and angles to test the validity of these model predictions in this relevant altered gravity regime. Until then, the model predictions, extrapolated to hypo-gravity, can be used as a reasonable preliminary estimate of static roll tilt perception.

The modified models were also simulated for static pitch tilt in hypo-gravity. The models predict a sensation of being pitched nose down relative actual pitch angle. Note this effect in hypo-gravity is opposite of that in hyper-gravity where the perception is pitch nose up relative to actual orientation. Due to the pitched up orientation of the utricular plane, the modified models make a peculiar prediction for extreme hypo-gravity levels (e.g., 0.05 G): at small pitch nose up orientations (e.g., +5°) both models predict a pitch nose-down perception (in our example, approximately −7° for the modified observer model and −4° for the modified utricular shear model, see Figure 7). Thus, the *direction* of pitch tilt can be misperceived in hypo-gravity. Note that for roll tilt in altered gravity the misperceptions are only *gain* errors (overestimation in hyper-gravity and underestimation in hypo-gravity), while direction is correct. A similar direction error is predicted for pitch tilt in hyper-gravity except it occurs for small pitch nose down tilts being misperceived as pitch nose up. To our knowledge static pitch tilt perception in hypo-gravity has not been quantified. Again, the modified models' predictions can be used as initial estimates for static pitch tilt in hypo-gravity.

There is some previous evidence (De Winkel et al., 2012) that at small hypo-gravity levels, the magnitude of gravity is too small to be used as a reference. Beyond this level, in the prior experiment the SVV generally aligned with the body longitudinal axis, as is common in microgravity. The threshold at which gravity is no longer used as a reference for perceptual orientation was seen to vary substantially among subjects, but on average was 0.3 G's (De Winkel et al., 2012). The gravity magnitude threshold effect is not present in the current modified model simulations. In the modified observer model, as long as the magnitude of gravity is >zero, near accurate perceptions are predicted at upright and 90° of roll tilt, while acute angles result in underestimation. The previously proposed concept of an “idiotropic vector” (Mittelstaedt, 1986, 1989; Vingerhoets et al., 2009), which drives perceptions toward the body longitudinal axis, could be added to the modified observer model to capture the low hypo-gravity threshold effect when appropriate.

### Application of the models for astronaut orientation perception

These novel models (modified utricular shear and modified observer) quantitatively match available tilt perception data in altered gravity. These advancements provide a substantial added capability for mathematical models of orientation perception. The modified utricular shear model provides a simple, one-equation prediction of static roll or pitch tilt in altered gravity. The modified observer model is more complex to evaluate, but while here we only simulated it for *static* tilts, it is capable of simulating *dynamic* motion profiles that involve sensory integration between otolith and semicircular canal cues. While previous models were either limited to static tilts (Schone, 1964; Correia et al., 1968; Mittelstaedt, 1983a; Dai et al., 1989; Bortolami et al., 2006) or 1 Earth G environments (Borah et al., 1988; Merfeld et al., 1993; Holly and McCollum, 1996; Glasauer and Merfeld, 1997; Haslwanter et al., 2000; Merfeld and Zupan, 2002; Angelaki et al., 2004; Laurens and Droulez, 2007; Vingerhoets et al., 2007; Macneilage et al., 2008; Selva and Oman, 2012), the modified observer model extends dynamic orientation perception models to altered gravity environments. In fact the modified observer model has been validated for perception of *dynamic* roll tilt in hyper-gravity (Clark et al., 2015). Future experiments are required to further validate predictions for dynamic perceptions in altered gravity. The observer model could be used to predict astronaut perceptions in an altered gravity environment, such as the moon or Mars, during complex motions, such as vehicle landing profiles.

However, there are a few limitations. First, the models assume the simulated subject has normal vestibular function (i.e., is adapted to a 1 Earth G environment). Yet, astronauts in microgravity undergo sensorimotor reinterpretation and adaptation (Young et al., 1984; Parker et al., 1985). Thus, an astronaut's orientation perception when landing on the Moon (~1/6 G) is likely to be affected by the three or more days of microgravity exposure during transit. These models do not attempt to capture prior adaptation to microgravity or any other altered gravity environment. Given the lack of quantitative data for orientation perception after microgravity adaptation, it would be difficult to validate any potential implementations of capturing this process in either of the modified models.

Second, while the modified observer model fits the available data well for roll and pitch tilt perception in hyper-gravity, it has not been validated for more complex motions or other aspects of orientation perception. Specifically the modified observer model has not been validated for (1) yaw rotation or azimuth perception in altered gravity, (2) translation perception in altered gravity, and (3) cases of visual-vestibular interaction in altered gravity.

Interestingly the modified observer model predicts an illusory perception of linear acceleration in hyper-gravity corresponding to vertical translation. The unmodified observer model also makes this prediction in hyper-gravity. This is the result of the presumption that the CNS utilizes an internal model of the physical law $\hat{\vec{a}}=\hat{\vec{f}}-\hat{\vec{g}}$ while assuming $\left|\hat{\vec{g}}\right|=1$. In hyper-gravity the magnitude of the estimated GIF $\left(\hat{\vec{f}}\right)$ is >1, but the magnitude of the estimate of gravity is fixed to 1 such that the excess magnitude is attributed to an estimated linear acceleration. Yet in post-experimental debrief subjects did not report illusory sensations of translation. These effects may have been quenched by subject knowledge of the device limitations (i.e., centrifuge cab could not translate) or non-vestibular cues that are not included in the observer model (e.g., proprioceptive or somatosensory cues). A similar illusory linear acceleration is also predicted in hypo-gravity, however the direction is opposite.

The Newman (2009) version of the observer model included pathways for visual cues and was able to mimic perceptions from many visual–vestibular interaction paradigms. The current observer model includes those pathways but deactivates them to simulate perceptions in the dark. The visual pathways can be activated and the modified observer model can predict perceptions for visual-vestibular paradigms in altered gravity. However, to our knowledge there is not a quantitative experimental dataset upon which to validate any of these predictions.

### Conflict of interest statement

The authors declare that the research was conducted in the absence of any commercial or financial relationships that could be construed as a potential conflict of interest.
