# Supplementary material for: Modeling human perception of orientation in altered gravity
Source: Front Syst Neurosci. 2015 May 5;9:68. doi: 10.3389/fnsys.2015.00068 (PMC4419856; doi:10.3389/fnsys.2015.00068)
Supplement: Supplementary file 1 [file Presentation1.PDF]

1. Appendix: Observer model details

1.1. Observer model review

The observer model has been published elsewhere (Merfeld et al., 1993;Merfeld and Zupan, 2002;Newman, 2009), but a summary is provided here. The model is a canal-otolith interaction model that has been extensively validated for several illusory perceptual paradigms in 1 Earth G, including Earth-vertical yaw rotation, linear acceleration, off-vertical axis rotation (OVAR), post-rotatory tilt, and the Coriolis cross-coupled illusion. We utilized the vestibular pathways of the Newman version of the model (2009), which is shown schematically in Figure A1.

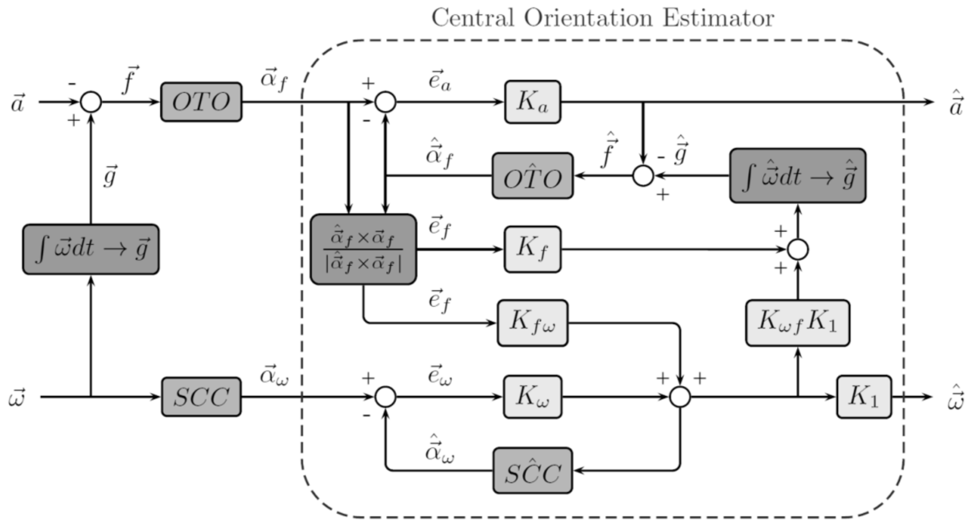

Figure A1: Schematic of the observer model.

The inputs to the model are three-dimensional linear acceleration ($\vec{a}$) and angular velocity ($\vec{\omega}$). Given an initial gravity vector ($\vec{g}_0$), angular rotations will yield a change in the orientation of gravity ($\vec{g}$) which is accounted for by rotational kinematics ($\int \vec{\omega}dt \rightarrow \vec{g}$), which are implemented using quaternion integration. The difference between linear acceleration ($\vec{a}$) and gravity ($\vec{g}$) yields the net gravito-inertial force (GIF) ($\vec{f}$). As in previous papers, the GIF is the input stimulation to the otolith organs (OTO), which are modeled with a 3x3 identity matrix (i.e., for simplicity, the earlier models ignored any dynamics of otolith transduction (Fernandez and Goldberg, 1976b;c;Fernandez and Goldberg, 1976a) because the high-frequency dynamics was shown to have little impact on model predictions). The otolith afferent response ($\vec{\alpha}_f$) thus is assumed to faithfully encode the GIF. Similarly, angular velocity is the input stimulation to the semicircular canals (SCC), which are also modeled with a 3x3 diagonal transfer function matrix, where each diagonal element is given in Equation A1 below.

Equation A1

$$\frac{\alpha_\omega(s)}{\omega(s)} = \frac{s^2}{(s + 1/\tau_d)(s + 1/\tau_a)}$$

Equation A1 relates the scalar angular velocity along one of the three axes (ω) to the semicircular canal afferent response along the same axis (α_ω) using the standard Laplace transform variable (s). The dominant time constant (τ_d) of the canals was set to 5.7 seconds and another "adaptation" time constant (τ_a) was included and set to 80 seconds, based upon neural recordings in squirrel monkeys (Fernandez and Goldberg, 1971;Goldberg and Fernandez, 1971). In addition the model utilizes a simple low pass filter with a cutoff frequency of 2 Hz for both the canal and otolith models to help ensure numerical stability. The value of the cutoff frequency has a negligible impact upon the simulation results.

In the model, it is assumed that the CNS neural networks have learned the normal relationships between head orientation and motion and the corresponding semicircular canal and otolith afferent responses. These networks are represented as an "internal model" based "observer" that continually evaluates vestibular sensory input and estimates head orientation and movement. The model assumes that the internal models of both the body ($\int \hat{\vec{\omega}} dt \rightarrow \hat{\vec{g}},\ \hat{\vec{f}} = \hat{\vec{g}} - \hat{\vec{a}}$) and sensory dynamics ($O\hat{T}O,\ \ S\hat{C}C$) are accurate (i.e. represent the physical laws and vestibular sensor dynamics), so no additional free parameters are introduced. As seen in Figure A1, the integration of perceived angular velocity to yield the perceived direction of gravity includes the contribution from the otolith pathways, thus perceived tilt is not necessarily the simple integration of perceived angular velocity. In the process of this integration, an internal estimate of the magnitude of gravity is applied. We assumed that since we spend the majority of our lives in a 1 Earth G environment that this internal magnitude is properly calibrated to 1 G. It should be noted that the assumed models of the sensory dynamics (i.e. identity matrix for the otolith and second order transfer function for the canals) are not critical to the model predictions as long as they are matched by the internal model of the sensory dynamics.

Two error vectors ($\vec{e}_a$ and $\vec{e}_f$) represent specific aspects of the difference between the actual otolith afference ($\vec{\alpha}_f$) and the expected otolith afference ($\widehat{\vec{\alpha}_f}$) based upon the internal model. The linear acceleration error ($\vec{e}_a$) is just the vector difference between the actual and expected afference, while the GIF rotation error ($\vec{e}_f$) represents the rotation required to bring the otolith measurement of the GIF into alignment with the internally estimated otolith measurement of GIF. There is only one angular velocity error vector ($\vec{e}_\omega$), which is simply the vector difference between the actual and expected afference. The mathematical formulations of these error calculations are given in Equations A2-A4.

Equation A2

$$\vec{e}_a = \vec{\alpha}_f - \hat{\vec{\alpha}}_f$$

Equation A3

$$\vec{e}_f = \frac{\vec{\alpha}_f \times \hat{\vec{\alpha}}_f}{\left|\vec{\alpha}_f \times \hat{\vec{\alpha}}_f\right|} cos^{-1}\left(\frac{\vec{\alpha}_f}{\left|\vec{\alpha}_f\right|} \cdot \frac{\hat{\vec{\alpha}}_f}{\left|\hat{\vec{\alpha}}_f\right|}\right)$$

Equation A4

$$\vec{e}_\omega = \vec{\alpha}_\omega - \hat{\vec{\alpha}}_\omega$$

Five constant scalar feedback gains (K_a , K_f , K_ω , $K_{f\omega}$, and $K_{\omega f}$) are used to "steer" the central estimates ($\hat{\vec{a}}$, $\hat{\vec{g}}$, and $\hat{\vec{\omega}}$) to values that minimize the sensory conflict errors ($\vec{e}_a$, $\vec{e}_f$, and $\vec{e}_\omega$). These five feedback gains serve as the only free parameters and represent how the CNS utilizes sensory information to make central estimates of orientation perception. A single set of free parameters is specified to predict perceptions from a wide range of motion paradigms. In addition, the scalar gain K_1 is a function of the angular velocity feedback gain ($K_1 = (K_\omega + 1)/K_\omega$) and is necessary to make the loop gain of the angular velocity feedback loop unity. In prior versions of the model, these scalar gains weight each of the three components (i.e. x, y, and z directions) of the error vectors equivalently. Actual roll (ϕ), pitch (θ), and yaw (ψ) orientation angles, as well as estimated angles ($\hat{\phi}$, $\hat{\theta}$, $\hat{\psi}$) are computed via Euler angle transformations from the actual ($\vec{g}$) and estimated ($\hat{\vec{g}}$) gravity vectors (e.g. for pure roll tilt $\theta = \text{atan}(g_y/g_z)$).

The model residual weighting parameters were set according to the values used by Vingerhoets (2007) and are shown in Table A1. This set of parameters is the only scheme validated exclusively on perceptual data as opposed to previous parameter sets based upon eye movement recording data (Merfeld et al., 1993;Haslwanter et al., 2000;Merfeld and Zupan, 2002).

Table A1: Model residual weighting parameters

Parameter	Value	Units
K_a	-4	Unitless
K_f	4	1/seconds
$K_{f\omega}$	8	1/seconds
K_ω	8	Unitless
$K_{\omega f}$	1	seconds

1.2. Conceptual modification to the observer model

We recently modified the observer model to enable it to predict the expected roll tilt overestimation in hyper-gravity (Clark et al., 2014). Details of that modification are provided here, specifically as it relates to the new simulations provided. We hypothesized that the modification should occur in the otolith pathways, thus the canal and canal-otolith interaction pathways were left unmodified. Specifically the modification was applied in the K_a pathway (see Figure A1). Conceptually, we hypothesized that the CNS treats otolith stimulation *in* the utricular plane differently than *out* of the plane (i.e. along the direction perpendicular to the utricular plane). This differential treatment is enabled by the relative weighting of the otolith sensory conflict signal differing depending upon its orientation relative to the utricular plane. It should be noted that the saccule transduces gravito-inertial stimulation approximately perpendicular to the utricular plane.

The proposed modification was loosely inspired by the utricular shear model (Schone, 1964), where it was assumed that the CNS estimates static orientation perception exclusively based upon the stimulation resulting from the shear forces acting in the plane of the utricular macula. Implementing Schöne's utricular shear theory in the Observer framework would imply that error

signals in the utricular plane are weighted by some finite constant and those out of the plane are ignored (i.e. weighted by zero). While our proposed modification does not aim to completely ignore stimulation out of the utricular plane, it does build upon the concept of weighting the signals differently as posited in the utricular shear theory. Ormsby and Young (1976) and Mittelstaedt (1983a;b) also hypothesized otolith signals are processed differently depending on their orientation relative to the utricular plane. However, as will be discussed later, none of these prior models posited that the differential treatment of otolith signals occur in the weighting of the sensory conflict signal.

1.3. Mathematical modification to the observer model

Previous versions of the observer model (Oman, 1982;Oman, 1991;Merfeld et al., 1993;Merfeld and Zupan, 2002;Zupan et al., 2002;Vingerhoets et al., 2007;Rader et al., 2009;Vingerhoets et al., 2009) all assumed the feedback gains were scalar. This assumes that the CNS processes sensory information from rotations around and accelerations along x-,y-, and z-axes equivalently. However, to implement the hypothesis that otolith sensory conflict signals are weighted differently depending upon the orientation relative to the utricular plane, we utilized a vector feedback gain. Specifically, the linear acceleration feedback (K_a) is extended to allow for different weightings of the linear acceleration sensory conflict error ($\vec{e}_a$) in the utricular plane versus out of the plane. All of the vectors in the model, including the linear acceleration error vector ($\vec{e}_a$), are in the head-fixed coordinate system defined above, with the x-axis aligned with the naso-occipital axis and the y-axis with the interaural axis. The error vector must first be rotated into a utricular plane-fixed coordinate system in order to differentially weight the components in and out of this plane. We assumed a simplified planar utricular macula defined by two axes: the x'-axis which is pitched up by $\theta_{utricle}$ from the head-fixed x-axis and the y'-axis which is aligned with the y-axis (interaural axis). Otolith stimulation in the x'- and y'-axes is in the utricular plane, while that in the z'-axis is out of plane. The rotation matrix to rotate linear acceleration feedback errors ($\vec{e}_a$) from the head-fixed coordinate system to the utricular plane system is given in Equation A5.

Equation A5

$$\begin{Bmatrix} e_{a_{x'}} \\ e_{a_{y'}} \\ e_{a_{z'}} \end{Bmatrix} = R(\theta_{utricle}) \begin{Bmatrix} e_{a_x} \\ e_{a_y} \\ e_{a_z} \end{Bmatrix} = \begin{bmatrix} \cos(\theta_{utricle}) & 0 & \sin(\theta_{utricle}) \\ 0 & 1 & 0 \\ -\sin(\theta_{utricle}) & 0 & \cos(\theta_{utricle}) \end{bmatrix} \begin{Bmatrix} e_{a_x} \\ e_{a_y} \\ e_{a_z} \end{Bmatrix}$$

Based upon morphology (Corvera et al., 1958;Curthoys et al., 1999), we fixed the value of $\theta_{utricle}$ to 30 degrees, however the specific value has no impact upon roll tilt simulations. As will be seen, this assumption does impact predicted pitch tilt perceptions in altered gravity. We further note that this angle would be expected to vary with peripheral anatomy of individual subjects. Once in the utricular plane coordinate system, we propose to weight the linear acceleration error vector ($\vec{e}_a$) by a vector of linear acceleration feedback gains ($\vec{K}_a$), such that the errors in the x'-y' plane are weighted differently than those out of plane (z' direction).

Equation A6

$$\vec{K}_a = \begin{Bmatrix} K_{a_{x'}} \\ K_{a_{y'}} \\ K_{a_{z'}} \end{Bmatrix} = \begin{Bmatrix} K_{a_u} \\ K_{a_u} \\ K_{a_{u\perp}} \end{Bmatrix}$$

The central estimate of linear acceleration is then finally rotated from the utricular plane-fixed frame back to the normal head-fixed coordinate system.

Equation A7

$$\begin{Bmatrix} \hat{a}_x \\ \hat{a}_y \\ \hat{a}_z \end{Bmatrix} = R^{-1}(\theta_{utricle}) \begin{Bmatrix} \hat{a}_{x'} \\ \hat{a}_{y'} \\ \hat{a}_{z'} \end{Bmatrix} = R(-\theta_{utricle}) \begin{Bmatrix} \hat{a}_{x'} \\ \hat{a}_{y'} \\ \hat{a}_{z'} \end{Bmatrix}$$

1.4. Fitting new free parameters of the observer model

The modification from Clark et al. (2014) to the model introduces one additional free parameter. Previously all of the components of the linear acceleration error ($\vec{e}_a$) were equally weighted by one feedback gain (K_a). In the modified model, each of the components of the linear acceleration error ($\vec{e}_a$) are now weighted by one of two independent feedback gains (K_{au} or $K_{au\perp}$). We chose to leave the linear acceleration feedback gain on errors *out* of the utricular plane ($K_{au\perp}$) at the nominal value of -4.0. The linear acceleration feedback gain on errors *in* the utricular plane (K_{au}) was then set to mimic the average static perception observed in a particular hyper-gravity case from our previous experiment (Clark et al., 2014): a 20 degree static roll tilt in 2 G's is perceived as approximately a 27 degree roll tilt. The K_{au} parameter was fit using just this one case instead of all the data for static roll tilt perception in hyper-gravity for two reasons: 1) We aimed to avoid over fitting. If the model fit to one static roll tilt angle could explain the data at other roll tilt angles it would provide greater confidence that the model can be applied across a wide range of angles in hyper-gravity. 2) "Evaluation" of the model for each static roll tilt angle requires a full simulation and thus an iterative "optimization" procedure is very computationally intensive.

Adjusting the utricular plane linear acceleration feedback gain (K_{au}) relative to the out of plane gain ($K_{au\perp}$) can produce over or underestimation in static perception of tilt in hyper-gravity. Of particular interest are the cases when the utricular plane linear acceleration feedback gain is greater than the out of plane gain ($K_{au} > K_{au\perp}$), which predict overestimation in the 2 G simulation. To mimic the amount of overestimation observed in our example case, the utricular plane linear acceleration feedback gain (K_{au}) must be approximately -2.2. Given limited physiologic and psychometric data, prior models only fit the feedback gain parameters to the nearest integer. To keep with this prior practice, we use a value of -2.0. A summary of the modified parameters in the model is provided in Table A2. In each of the following simulations, the same structure and feedback parameters (Tables A1 and A2) were held constant.

Table A2: Modified observer model parameters.

Parameter	Value	Units
K_{au}	-2.0	Unitless

$K_{au\perp}$	-4.0	Unitless
$\theta_{utricle}$	30	degrees

1.5. Observer model implementation and simulations

We implemented the model in MATLAB – Simulink 2012a (The MathWorks) software package. The Simulink model was simulated using a variable time-step fourth order Runge-Kutta differential equation solver (ode45 Dormand-Prince). Motion profile inputs were provided at 100 Hz. Nominally, simulations always start with the actual and perceived orientation upright (i.e. 0 degrees of roll and pitch tilt). Thus, to simulate the static tilts considered below motion profiles were created that dynamically tilted the simulated observer to the desired angle and then simulated 30 seconds with no dynamic motion (i.e. static tilt), after which the prediction was negligibly affected by the dynamic rotation profile (< 0.001 degrees) and thus can sufficiently be considered "static". The observer model's predicted orientation perception at 30 seconds after the end of the dynamic motion was taken as the *static* measures reported below.
